# Biological Control and Habitat Management for the Control of Onion Thrips, *Thrips tabaci* Lindeman (Thysanoptera: Thripidae), in Onion Production in Quebec, Canada

**DOI:** 10.3390/insects15040232

**Published:** 2024-03-27

**Authors:** Annie-Ève Gagnon, Anne-Marie Fortier, Carolane Audette

**Affiliations:** 1 Saint-Jean-sur-Richelieu Research and Development Centre, Agriculture and Agri-Food Canada, 430 Gouin Boulevard, Saint-Jean-sur-Richelieu, QC J3B 3E6, Canada; carolane.audette@agr.gc.ca; 2 Compagnie de Recherche Phytodata Inc., 291 Rue de la Coopérative, Sherrington, QC J0L 2N0, Canada; afortier@phytodata.ca

**Keywords:** onion thrips, pest management, flower strips, straw mulch, biological control, exclusion net, kaolin, mineral oil, habitat manipulation

## Abstract

**Simple Summary:**

Onion thrips are a major threat to onion crops, causing damage and yield loss. This study tested various pest control methods, including habitat manipulations, biological control agents, and physical barriers, in experimental and commercial field conditions with the aim of managing thrips populations. The results showed that modifying habitats with straw mulch and flower strips effectively reduced thrips numbers and improved onion yields. However, the use of exclusion nets had a negative effect on yields, and the other control methods produced results similar to those obtained for the untreated control. Notably, the use of biological agents on their own did not provide effective thrips control. This research provides valuable insights for sustainable and effective pest management in onion production.

**Abstract:**

Onion thrips (*Thrips tabaci*) can pose a significant threat to onion crops, causing leaf damage, reduced bulb size and quality, and yield loss during severe infestations. Conventional insecticide use has been the primary method for managing this pest species, but the efficacy of this approach is inconsistent. Furthermore, emerging pest resistance is a growing concern in some regions. This two-year field study aimed to assess the effectiveness of several pest management strategies in controlling onion thrips populations and limiting their impact on onion yields. The strategies tested consisted of habitat manipulations (including flower strips and straw mulch), biological control agents (*Stratiolaelaps scimitus*, *Neoseiulus cucumeris*, *Amblyseius swirskii*, and *Beauveria bassiana*), as well as physical barrier control methods (exclusion nets, kaolin, and mineral oil). Habitat manipulation techniques, particularly the use of flower strips, reduced thrips populations by up to 50% and increased onion yields by 25%. In contrast, exclusion nets had a detrimental effect on onion yields, and the other alternative control methods produced results comparable to those obtained for untreated controls. When used alone, biological control agents were not effective at maintaining thrips populations below economically damaging levels. This study offers valuable insights into effective and sustainable pest management practices for the onion industry.

## 1. Introduction

Dry onions are grown on 5700 hectares in Canada, including 2250 ha in Quebec (39.4%) and 2340 ha in Ontario (41.1%) [1], and Canadian production is valued at $157 million annually [2]. Onion thrips, *Thrips tabaci* Lindeman, 1889 (Thysanoptera: Thripidae), is a common pest of onion crops worldwide [3]. These insects feed on the leaves of onion plants, causing their distortion and discoloration, resulting in a decrease in bulb size and quality [4]. In severe thrips outbreaks, bulb yields may be substantially reduced [5]. Injuries to onion leaf tissue also create entryways for bacterial and foliar pathogens [6,7]. *Thrips tabaci* is the primary vector of Iris yellow spot virus (IYSV), which can cause serious damage to onion plants [8] and was recently reported in Canada [9].

Onion thrips overwinter as adults, buried in residues in onion and other crop fields as well as in weeds. Thrips begin laying eggs in the spring on the first available host plants [10]. Higher adult dispersal activity is observed when temperatures are warm (between 21 and 28 °C) and wind speed is low [11]. After feeding on onion leaves, the larvae drop to the ground to pupate [3]. Since thrips generally complete their life cycle within two weeks during the summer, several overlapping generations may occur during a given production season [12].

Insecticides are frequently used to reduce thrips infestations, even during periods of relatively low population density, because outbreaks are difficult to predict. Management of thrips populations through chemical means poses challenges given that females deposit their eggs within the leaf tissues, larvae hide among inner leaves, and pupae are concealed in the soil [4]. Furthermore, thrips resistance to certain insecticide active ingredients has been documented in New York State and Ontario [13,14,15]. The correlation between thrips density and yield loss is variable, and this variability may be explained in part by various factors such as plant variety, onion phenological stage, and water stress [5]. In addition to these considerations, onion producers are looking for alternatives to insecticides for maintaining the sustainability of onion production while protecting biodiversity on the farm.

A number of studies have evaluated the potential of biological control agents against thrips in vegetable crops. For example, applications of the entomopathogenic fungus *Metarhizium anisopliae* (Metschn.) Sorokin against *T. tabaci* in Kenyan onion crops significantly reduced damage to an extent comparable to insecticide treatments [16]. In greenhouse trials, the use of the predatory mites *Stratiolaelaps scimitus* (Womersley) and *Neoseiulus barkeri* Hughes against *T. tabaci* in cucumbers reduced thrips populations by up to 76% [17]. Local natural enemies, including predatory bugs (*Orius* sp.), ladybugs, lacewings, hoverflies, and predatory thrips, provide effective and complementary control of onion thrips [18,19]. The combined use of biological control agents that attack both the foliar (larvae and adults) and ground (pupae) stages of thrips is likely to increase effectiveness. In a compatibility study of control agents against the western flower thrips (*Frankliniella occidentalis*), Saito and Brownbridge [20] showed in controlled conditions that combining the use of the ground predators *Dalotia coriaria* (Kraatz), *S. scimitus*, and *Gaeolaelaps gillespiei* Beaulieu with the entomopathogenic fungi *M. anisopliae* and *Beauveria bassiana* (Bals.) significantly increased control, achieving a reduction of 90% in the thrips population compared with less than 60% when the different biological control agents were used individually.

The presence of flower strips around a field can also attract natural enemies of thrips (e.g., hoverflies, lacewings, and parasitoids) that feed on nectar or pollen. In addition to providing an alternative food source, flower strips provide shelter for these biological control agents [21]. By maintaining populations of natural enemies near the crop, floral plantings can help to quickly manage future damage caused by pests when their numbers increase in the crop [22,23]. Alcalá Herrera et al. [24] reported that flower strips along cabbage rows attracted more predators and parasitoids, resulting in lower pest densities (lepidopterans and aphids) than in the control without flower strips. Little research has been conducted on the use of flower strips for managing onion thrips in dry onion cultivation. The use of straw mulches, another type of habitat manipulation, generally creates an environment that is more structurally complex than bare soil and also provides a cooler and wetter microclimate [25]. Mulches provide new niches and refuges that contribute to more diverse arthropod communities including predators and granivores [26,27]. Another positive effect of mulches in thrips management has been demonstrated through the use of UV-reflective mulches, which reduce thrips populations by hindering the insects’ ability to recognize and colonize their hosts [28,29,30,31].

Physical pest management strategies that disrupt insect colonization or the insects’ ability to pierce the leaf surface with their mouthparts can be useful in slowing pest emergence in crops. Kaolin-based particle films have been found to be effective in suppressing several plant-feeding and virus-vector arthropods and in reducing plant stress in various crops without affecting plant photosynthesis or productivity [32,33,34,35]. This approach yields several advantageous outcomes, including diminished oviposition, reduced hatching, altered feeding preferences, prolonged development time, increased mortality rates, and reduced abilities to detect host plants [36]. Mineral oil is another product that is commonly used to deter pest populations, especially disease-vectoring species like aphids (e.g., ref. [37]) or psyllids (e.g., ref. [38]). Mineral oils act by forming a thin film on the surfaces of plant leaves, stems, and fruits, and are considered contact insecticides that disrupt respiration and membrane function, and in some cases alter feeding behavior [39]. Finally, exclusion nets are becoming an increasingly popular tool in agriculture for minimizing insect pest damage [40]. These nets act as physical barriers, preventing insects from reaching crops and therefore significantly reducing their impact [41].

Our objective was to evaluate alternative pest management strategies involving different approaches: (i) biological control agents, used alone or in combination; (ii) habitat manipulation with the use of flower strips and straw mulches; and (ii) products or strategies such as kaolin, mineral oil, or nets that are aimed at reducing plant colonization by thrips. The purpose of this study was to identify the most promising pest management strategies and then propose a set of measures that could be combined to adequately control thrips densities while reducing or eliminating the use of chemical insecticides.

## 2. Materials and Methods

### 2.1. Experimental Sites

Field experiments were conducted in 2021 and 2022 at the Agriculture and Agri-Food Canada (AAFC) experimental farm located in Sainte-Clotilde-de-Châteauguay, QC, Canada (AAFC site: 45.17 N, −73.68 W), and on two commercial sites located in the Les-Jardins-de-Napierville regional county municipality (Site 1: 45.20 N, −73.36 W; Site 2: 45.13 N, −73.52 W). The AAFC site and Site 2 were characterized by organic soils, specifically muck soil, in contrast to Site 1, which had mineral soil. The onion cultivars used in this experiment were as follows: Trailblazer at the AAFC site, Patterson at Site 1, and Cartier at Site 2. All of them were seeded and transplanted on similar dates (Supplemental Table S1). The experimental design varied between years and sites; however, in each case, all treatments were repeated four times using a randomized block design or a split-plot design (see each site description hereafter). Each plot consisted of three beds, 2 m wide, for a total width of 6 m per plot, and 10 m long. Within each bed, four double rows of onions spaced 0.4 m apart were seeded at a rate of 30 onions per meter. Plots were spaced 4 m apart and blocks were spaced 10 m apart (unless otherwise specified). Onions were irrigated and fertilized as needed, and disease and weed management followed local recommendations [42] (Supplemental Table S2). No insecticide applications were made in the experimental plots apart from a commercial control treatment.

### 2.2. Pest Management Treatments

#### 2.2.1. 2021 AAFC Site

Three different habitat management methods were tested at the AAFC site: flower strips, straw mulch, and bare soil (as a control). The flower strip treatment consisted of two strips 4 m by 20 m placed on the opposite sides of each plot. Flower strips were composed of a mix of 10 indigenous species (*Agastache foeniculum* [Pursh] Kuntze, *Asclepias syriaca* L., *Astragalus canadensis* L., *Desmodium canadense* (L.), *Eupatorium maculatum* L., *Heliopsis helianthoides* (L.), *Monarda fistulosa* L., *Rudbeckia hirta* L., *Symphyotrichum novae-angliae* (L.), and *Verbena hastata* L.) combined with a 1.8 m × 20 m strip of coriander (*Coriandrum sativum* L.). All indigenous flowers were planted in 2018 and minimally maintained (mowing every fall and weeding in spring) until the experiment began. The coriander was sown on 15 May 2021. The straw mulch consisted of dry straw of switchgrass (*Panicum virgatum* L.) applied between rows at the three-leaf onion stage, at a rate of 7 tonnes/ha. The bare soil treatment with no additional interventions served as a control.

Within each habitat management treatment, four different pest management strategies were tested using a split-plot design, with the treatments in the sub-plots consisting of the following: (1) soil biological control agents, (2) foliar biological control agents, (3) combination of biological control agents, and (4) insecticide treatment (commercial control). The soil biological control treatment consisted of using an indigenous predatory mite, *Stratiolaelaps scimitus* (Womersley), that feeds on thrips pupae and nymphs [17,43]. The initial application was made at the first sighting of thrips in the onion field, at a rate of 50 mites/m^2^, and two additional applications were made subsequently at the same rate (Supplemental Table S3). The foliar biological control treatment consisted of using *Neoseiulus cucumeris* (Oudemans), a predatory mite that feeds mainly on the first instars of thrips [44]. These mites were inoculated with spores of *Beauveria bassiana* (Bals.) which acted as a fungal vector [45,46]. This fungus is known to infect and kill thrips, but it is innocuous for *N. cucumeris* [47]. As in the case of soil mites, the first application of *N. cucumeris* + *B. bassiana* was performed at the first sighting of thrips, but the rate was adjusted according to thrips density, i.e., depending on whether the thrips density threshold (1 thrips/leaf) had been reached or had not (50 mites/m^2^ if under threshold, 150 mites/m^2^ if threshold reached) (Supplemental Table S3). The combination of biological control treatments involved introducing two predatory mite species, *S. scimitus* and *N. cucumeris*, inoculated with *B. bassiana,* and applied at the same time and rate as mentioned previously. Recommendations for release rates and timing of all the biocontrol agents were suggested by Anatis Bioprotection Inc. (Saint-Jacques-le-Mineur, QC, Canada). The biological control inocula, supplied by the same company in vermiculite, were manually distributed in the plots. The insecticide treatment consisted of a conventional approach whereby the first application was made at the first thrips sighting and spraying was repeated weekly by rotating the insecticides used, if the thrips density reached the threshold level (Supplemental Table S3).

#### 2.2.2. 2022 AAFC Site

The field trial conducted at the AAFC site in 2022 involved the following treatments for comparison: (1) control without insecticide; (2) exclusion net; (3) straw mulch; (4) straw mulch + flower strips; (5) flower strips; (6) kaolin; (7) mineral oil; (8) *A. swirskii*; (9) *B. bassiana*; and (10) *A. swirskii* + *B. bassiana*. The control treatment in 2022 involved no pesticide interventions, in contrast to 2021 where insecticides were applied, except for the application of herbicides and fungicides as needed (Supplemental Table S4). Building on the results obtained in 2021, we have adjusted the conventional control treatment to consist of no phytosanitary interventions. This choice allows us to truly observe the reduction in thrips density associated with each treatment and its direct impact on yield. Exclusion nets were constructed of 0.25 × 0.35 mm mesh (70 g/m^2^; Dubois Agrinovation, St-Rémi, QC, Canada) and were installed at the four-leaf stage of onions or directly after planting in the case of Site 1. Each plot was protected by an 8 × 10 m net placed over metal hoops (three per bed) and secured in place with 10 rock bags. The netting was removed for short periods when fungicide treatments or weeding were necessary and then promptly reinstalled, depending on re-entry time constraints. The straw mulches and flower strips used in 2022 were the same as those described for 2021, except that flower strips plots were physically separated (>200 m) from the rest of the experiment to prevent any potential spillover of natural enemies attracted to the flowers. The first application of kaolin (Surround^®^ WP, NovaSource-Tessenderlo Kerey Inc., Phoenix, AZ, USA) was performed at the 2–3 leaf stage of onions and then repeated every 5 to 10 days when an infestation of thrips was observed. The initial two applications were carried out at a concentration of 25 kg/500 L of water/ha, and the subsequent applications at a lower concentration, that is, 12.5 kg/500 L of water/ha. The mineral oil treatment (SuffOil-X^®^, BioWorks Inc., Victor, NY, USA) was applied according to the same schedule as for kaolin, using a rate of 6.5 L/1000 L of water/ha according to the label instructions. In 2022, we used the predatory mite *Amblyseius swirskii* (Athias-Henriot) as a foliar biological control agent; it was supplied by Anatis Bioprotection Inc. Inoculation of *A. swirskii* began as soon as the first thrips were observed in the onion plots (Supplemental Table S4). The initial introduction was done at a rate of 50 mites/m^2^ and subsequent applications were conducted weekly. If the threshold of 1 thrips/leaf was reached, the rate was increased to 100 mites/m^2^, otherwise it was maintained at 50 mites/m^2^. The bioinsecticide *B. bassiana* (BioTitan^®^ WP, Anatis Bioprotection Inc.) was used and applied under the same conditions as in 2021 (Supplemental Table S4). The combination of biological control agents involved the addition of *A. swirskii* inoculated by *B. bassiana* at the same time and rate and under the same conditions as described above for the biological control agents that were inoculated separately.

#### 2.2.3. 2021 Sites 1 and 2

At Sites 1 and 2, different pest management treatments were tested without comparing habitat management methods, i.e., all plots at these sites were considered as bare ground treatment. Treatments at these sites were exactly the same as for 2 to 4 described above, with three variants added: a control treatment without insecticide, a treatment combining *N. cucumeris* (without entomopathogenic spores of *B. bassiana*) and *S. scimitus* (used at the same rate as described above), and a treatment using *B. bassiana* alone (BioCérès^®^ WP, Anatis Bioprotection Inc.). This product was applied at a concentration of 6 g/L and at a rate of 500 L/ha of water, with spraying done in the evening to prevent the detrimental effects of UV on spores [48]. Applications were started upon the initial sighting of thrips in the field and were repeated weekly if the threshold of 1 thrips/leaf was reached (Supplemental Table S3). Insecticides were applied on a weekly basis as necessary when the threshold of 1 thrips/leaf was met (Supplemental Table S3).

#### 2.2.4. 2022 Site 1

The treatments applied at Site 1 in 2022 were identical to those described for AAFC 2022, with the exception that flower strips were not implemented at this site.

### 2.3. Data Collection

Onion monitoring was conducted in each plot every week, starting at the 2–3 leaf stage. Two randomly selected monitoring zones, each consisting of five consecutive onion plants, were carefully examined to determine the number of thrips adults and larvae per plant. The identification of *T. tabaci* was done visually in the field based on adult size and pattern on the abdominal segments. The number of leaves per plant and the average phenological stage were recorded weekly for each plot until more than 50% of the onions had lodged. Mean thrips density per leaf (adults and juveniles combined) was calculated after each monitoring visit to determine if the threshold of 1 thrips per leaf had been reached, indicating the need for treatment applications. This threshold of 1 thrips per leaf was recommended by agronomic consultants in the province of Quebec, Canada [49].

At the end of the season, the onions in a 2 m linear section of the two middle rows in each plot (4 m linear section per plot) were carefully uprooted from the soil and set aside in containers to dry on the ground for approximately three weeks. Once the drying process was complete, the size and weight of the onions were measured. The total number of onions per plot was determined, and the onions were classified into six grades based on their diameter: downgraded (<5.08 cm), 5.08–5.71 cm, 5.72–6.35 cm, 6.36–6.98 cm, 6.99–7.62 cm, and >7.62 cm. Onions displaying disease symptoms were categorized as downgraded. The marketable weight was calculated by deducting the weight of the downgraded onions from the total weight of the onions in each plot.

### 2.4. Data Analysis

The statistical analysis was conducted separately for each year (2021 and 2022) and site (AAFC site, Site 1 and Site 2). Thrips density was plotted using the number of thrips per leaf. This was facilitated by the consistent number of leaves across treatments, enabling comparison of leaf density measurements. Thrips abundance was also compared among treatments using a cumulative thrips–days (CTD) index based on the following formula: CTD = Cum_j_ + [([c_j_ + c_k_]/2)(t_j_ − t_k_)], where *j* and *k* are two consecutive monitoring events, *t* represents the monitoring date, Cum_j_ is the cumulative number of thrips–days per plant at time *j*, and *c* is the number of thrips per plant [5]. For each site–year experiment, analyses of variance (ANOVAs) were performed using the aov function with “treatment” as the fixed factor and “block” as the random factor. Shapiro–Wilk and Levene’s tests were conducted beforehand to confirm that the data met the assumptions of normality and homogeneity of variance, respectively, for the ANOVA postulates. Differences among treatment means were determined using Tukey’s honestly significant difference (HSD) tests, and the ‘HSD.test’ function in the ‘agricolae’ package was used. For the AAFC site, a split-plot design was applied with habitat manipulation being the whole-plot factor and the biological control methods being the split-plot factor. A mixed model approach was performed using a lmer function with habitat manipulation and biological control methods considered as fixed factors and plot considered as the random effect in order to account for the whole-plot error and the split-plot error. Multiple comparisons were performed using a Tukey test in the multicomp package, and contrasts between biological control treatments were performed using the emeans function. R software (version 4.2.2) was used to perform all statistical analyses [50].

## 3. Results

### 3.1. Abundance of Onion Thrips

**Figure 1:**
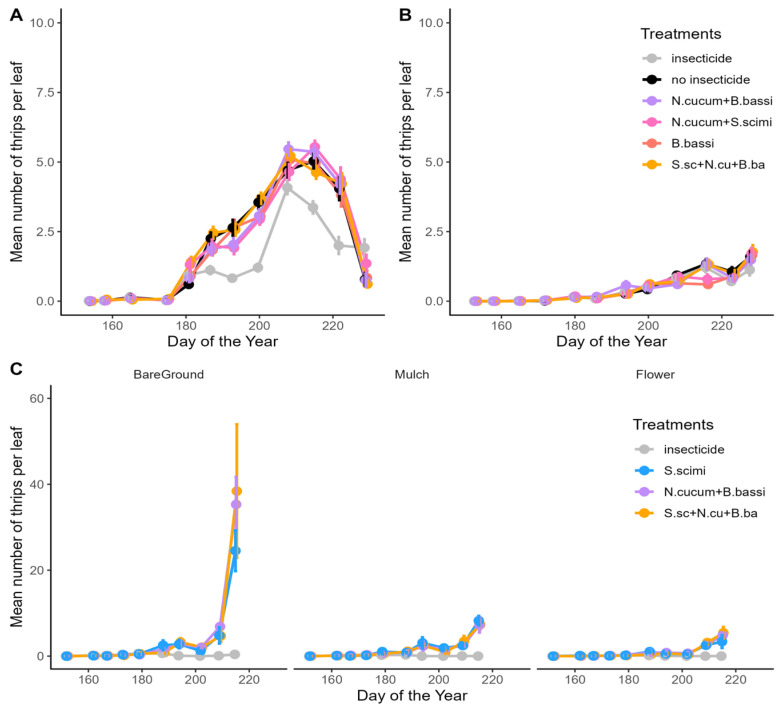
Seasonal variation in the mean number of *Thrips tabaci* per leaf (±SE) in onion fields in 2021 at (**A**) Site 1; (**B**) Site 2; and (**C**) AAFC site. Panels A and B share a common legend.

**Figure 2:**
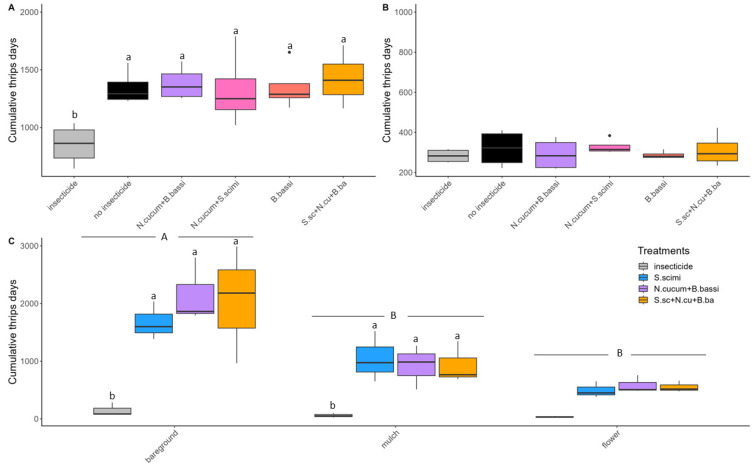
Cumulative number of thrips-days per plant for the different *Thrips tabaci* control methods compared in 2021 at (**A**) Site 1; (**B**) Site 2; and (**C**) AAFC site. Means with the same letter among the treatments (lowercase) or the habitat management methods (uppercase) are not significantly different (*p* > 0.05). Black dots represent outliers (i.e., exceptionally diverging values).

In 2021, thrips density was particularly high at the AAFC site, reaching a maximum mean density of 38.4 thrips per leaf, in comparison with a maximum mean density per treatment of 5.5 and 1.8 thrips per leaf, respectively, at Site 1 and 2 (Figure 1). The number of thrips per plant, as well as the number of adult or juvenile thrips per leaf, also exhibited the same trends across the different treatments as the overall number of thrips per leaf (Supplemental Figures S1–S3). The comparison of CTD values showed a significant difference between the insecticide treatment and the biological control methods at Site 1 (F_(5,15)_ = 10.42; *p* = 0.0002; Figure 2A), while no difference was found at Site 2 (F_(5,15)_ = 0.98; *p* = 0.4591; Figure 2B). At the AAFC site, both the habitat manipulation treatments (F_(2,24)_ = 22.52; *p* < 0.0001) and the biological control treatments (F_(3,24)_ = 15.82; *p* < 0.0001) were found to be significantly different in terms of CTD counts (Figure 2C). A higher number of thrips was observed in the bare soil treatment relative to the straw mulch (z value = −5.132; *p* < 0.0001) and flower strip treatments (z value = −3.819; *p* = 0.0005). This lower thrips abundance represents a substantial decrease of 50.8% in CTD in the straw mulch treatment and 72.3% in the flower strip treatments. The mean CTD values obtained for the biological control treatments did not differ significantly; however, they exceeded the values for the insecticide control on bare soil (*S. scimitus*: t-ratio = −5.24, *p* = 0.0002; *N. cucumeris* + *B. bassiana*: t-ratio = 6.88, *p* < 0.0001; *S. scimitus* + *N. cucumeris* + *B. bassiana*: t-ratio = 6.51, *p* < 0.0001) and the straw mulch (*S. scimitus*: t-ratio = −3.40, *p* = 0.0127; *N. cucumeris* + *B. bassiana*: t-ratio = 2.97, *p* = 0.0334; *S. scimitus* + *N. cucumeris* + *B. bassiana*: t-ratio = 3.01, *p* = 0.0306; Figure 2C). No significant differences in the mean CTD values were observed between the biological control treatment and the use of insecticides in the flower strips treatment (*S. scimitus*: t-ratio = −1.580, *p* = 0.4101; *N. cucumeris* + *B. bassiana*: t-ratio = 1.89, *p* = 0.2618; *S. scimitus* + *N. cucumeris* + *B. bassiana*: t-ratio = 1.78, *p* = 0.3111; Figure 2C).

**Figure 3:**
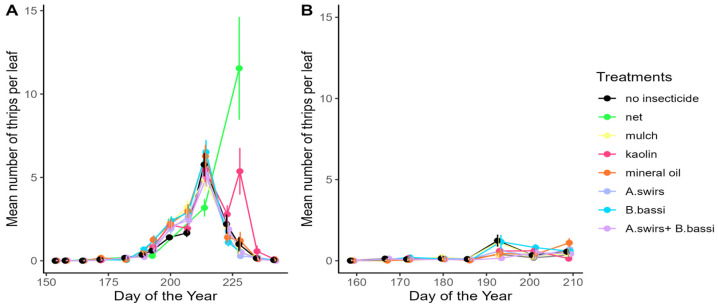
Seasonal variation of the mean number of *Thrips tabaci* per leaf (±SE) in onion fields in 2022 at (**A**) Site 1; (**B**) AAFC site. Panels A and B share a common legend.

**Figure 4:**
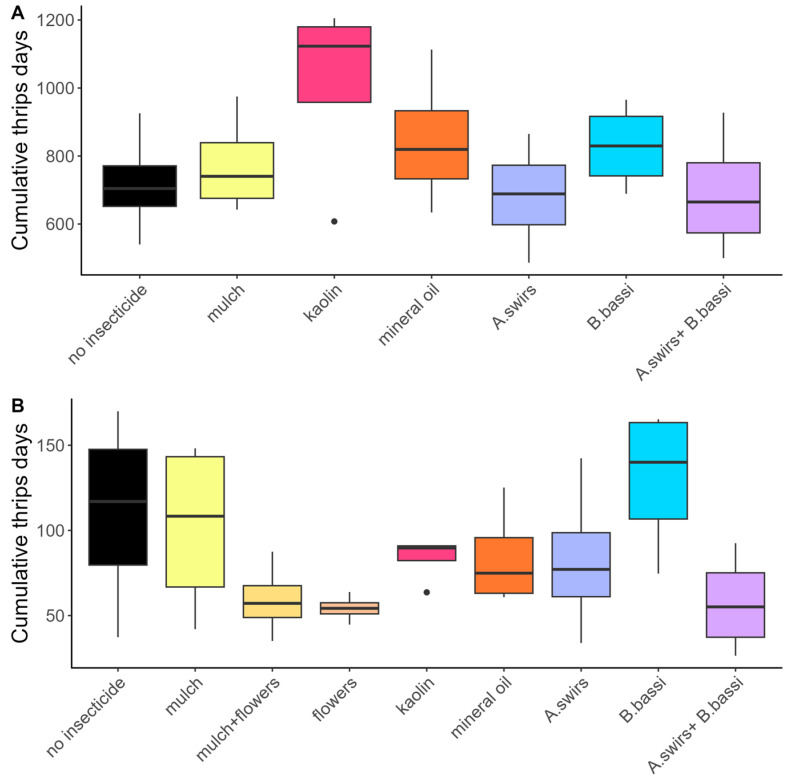
Cumulative number of thrips-days per plant for the different *Thrips tabaci* control methods compared in 2022 at (**A**) Site 1; (**B**) AAFC site. Black dots represent outliers (i.e., exceptionally diverging values).

In 2022, thrips density at the AAFC site was lower than in 2021, reaching a maximum mean of 1.2 thrips per leaf. Density at Site 1 was slightly higher than in 2021, with a mean of 6.5 thrips per leaf (Figure 3). The trends in the number of thrips per plant, as well as the number of adult or juvenile thrips per leaf, mirrored those observed for the number of thrips per leaf across various treatments in 2022 (Supplemental Figures S4–S6). Thrips density was particularly high on Site 1 in the treatment using exclusion nets, since the insects were able to enter inside the nets during weeding operations and multiply in the absence of control methods (no sampling of thrips was performed under the nets at the AAFC site). No significant difference in CTD was observed among the treatments at Site 1 (F_(6,18)_ = 1.71; *p* = 0.1770; Figure 4A) and the AAFC site (F_(6,18)_ = 1.99; *p* = 0.1201; Figure 4B) in 2022.

### 3.2. Yield and Onion Categories

**Figure 5:**
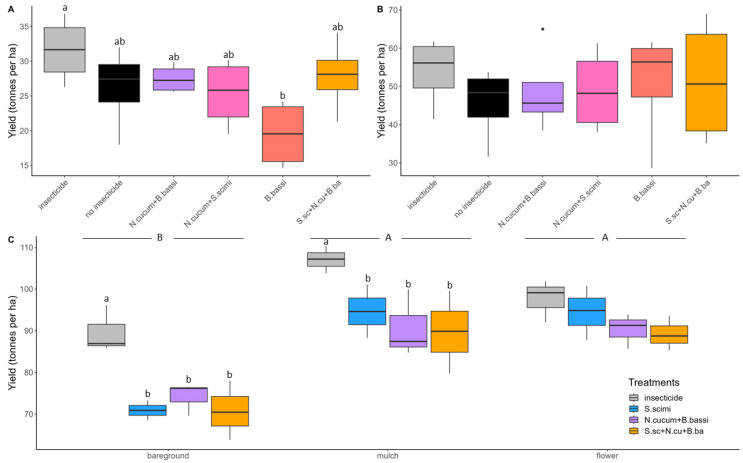
Mean onion yield (±SE) in 2021 at (**A**) Site 1; (**B**) Site 2; and (**C**) AAFC site. Means with the same letter among the treatments (lowercase) or the habitat management methods (uppercase) are not significantly different (*p* > 0.05). Black dots represent outliers (i.e., exceptionally diverging values).

Despite the significant reduction in thrips populations achieved through insecticide use in 2021, onion yields at Site 1 were consistent across all treatments, except for the treatment using *B. bassiana*, which had a much lower yield (F_(5,15)_ = 63.88; *p* = 0.0378; Figure 5A). No difference in yield was observed among the treatments at Site 2 (F_(5,15)_ = 0.65; *p* = 0.6656; Figure 5B), but thrips densities were low. For the AAFC site in 2021, habitat management (F_(2,18)_ = 48.89; *p* < 0.0001) and biological control treatments (F_(3,18)_ = 15.35; *p* < 0.0001) had significantly different effects on onion yield. Significantly higher yields were recorded for onions produced using straw mulch (z value = 3.376; *p* = 0.0021) and flower strip management (z value = 3.641; *p* < 0.0001; Figure 5C), with remarkable gains of 25.1% and 21.7%, respectively, relative to the control. Furthermore, yields were higher with insecticide use in comparison with the alternative biological control methods used on bare ground and the straw mulch treatments (Figure 5C). With regard to onion size, more downgraded onions were observed in the bare ground treatment compared to the straw mulch and flower strip treatments (Supplemental Table S5).

**Figure 6:**
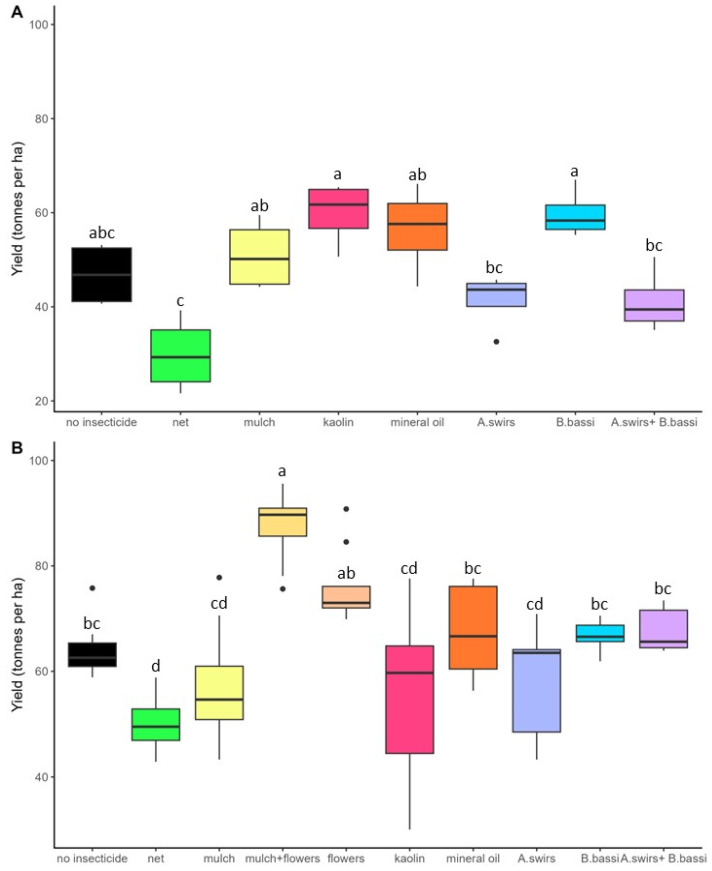
Mean onion yield (±SE) in 2022 at (**A**) Site 1; (**B**) AAFC site. Means with the same letter are not significantly different (*p* > 0.05). Black dots represent outliers (i.e., exceptionally diverging values).

In 2022, the different pest management treatments had a significant effect on onion yields at both Site 1 (F_(7,21)_ = 8.25; *p* < 0.0001; Figure 6A) and the AAFC site (F_(7,53)_ = 5.53; *p* < 0.0001; Figure 6B). The use of an exclusion net had a pronounced negative effect on onion yield at both sites under study. With regard to the other alternative control methods, only the straw mulch + flower treatment on the AAFC site was significantly different from the control without insecticide treatment. On Site 1, an upward trend in yield was observed for the kaolin and *B. bassiana* treatments; however, the yield was not significantly different from the treatment without insecticide. The size of the onions varied slightly among the treatments: the straw mulch + flower treatment produced more onions larger than 7.62 cm and the exclusion nets treatment produced smaller onions, including a high proportion of downgraded onions (Supplemental Table S6).

### 3.3. Correlation between Onion Yield and Insecticide Treatments

Insecticide treatments provided effective control of thrips across all trial sites in the present study. Yield is not always directly correlated with thrips density, and insecticide treatments that successfully control thrips populations are not consistently associated with increased yield. For example, the insecticide (Entrust) treatment at Site 1 in 2021 was not associated with improved yield, despite the marked decrease in thrips density. Conversely, an increase in yield was observed at the AAFC site, where thrips density was exceptionally high in 2021. When the relationship between cumulative thrips–days and onion yield at each site is examined, no correlation is found for Site 1 2021 (Supplemental Figure S7A,B). However, a strong correlation is observed for the AAFC site and Site 2 in 2021 (Supplemental Figure S1C). No correlation was observed in 2022 for the two sites under study owing to low thrips densities (Supplemental Figure S8A,B).

## 4. Discussion

This study examined the effectiveness of different pest management strategies based on thrips density and onion yield under field conditions. Biological control agents, used alone or in combinations, did not control thrips density efficiently, i.e., they did not reduce thrips density below the economic threshold of 1 thrips per leaf in either year of the study. The use of straw mulches and flower strips resulted in reduced thrips density, and higher onion yields were observed at one site in 2021. In 2022, exclusion nets had a negative effect on onion yield, while all the other alternative control methods did not significantly differ from the control without insecticide treatment in this regard. These findings suggest that management practices such as straw mulch and flower strips hold great promise from the standpoint of onion thrips control as well as higher yields and good onion quality. However, more research is needed to determine why biological control agents failed to control thrips densities under field conditions.

### 4.1. Flower Strips and Straw Mulch: Promising Techniques for Onion Thrips Control

Habitat manipulations are agronomic techniques that can increase biodiversity on farms and foster the biological control of pests [51]. Numerous studies have shown the potential benefits of using flower strips in vegetable crops [21,22,23]. For example, more parasitoids and predators have been observed in fields adjacent to flower strips, a finding that has been associated with reduced abundance of lepidopteran pests and the aphid *Brevicoryne brassicae* L. in cabbage production [24]. Our study reveals the promising potential that flower strips offer. In 2021, at the AAFC site, flower strip treatment was associated with thrips densities similar to those observed in plots where insecticide treatments were applied. This indicates that the addition of floral resources led to a reduction in thrips populations, as thrips numbers were much higher in plots without habitat manipulation. These effects could be attributable to the conservation of natural enemies in onion fields. However, surveys have found very low numbers of natural enemies, with less than 20 individuals in 2021 and 50 individuals in 2022, across all plots throughout the entire sampling period (Supplemental Table S7). The main groups of natural enemies found were hoverflies, predatory thrips, green lacewings, and ladybugs. Although hoverflies are not usually considered significant thrips predators, their presence in flower strips or straw mulch would likely favour their population growth and thrips consumption [52]. Onion plants offer limited shelter and few microclimate sites that can protect natural enemies from adverse weather conditions. Consequently, these beneficial insects likely travel between flower strips and the crop, serving as a connecting link or reservoir [53]. Another noteworthy aspect relates to the olfactory or visual confusion produced by flowers growing near onion plots [54]. *Thrips tabaci* is a species that locates its host plants through olfactory and visual stimuli [55,56]. However, it is not clear whether the activity of *T. tabaci* is disrupted by the addition of floral resources which mask the onion-specific olfactory signal, or conversely, whether thrips are more attracted to the flower scents and thus remain in the section of field where flower strips are established [57]. In our study, we also conducted weekly sampling in the flower strips throughout the growing season (June to August) using a beating net (Supplemental Figure S9). However, the number of thrips (all species combined) was low, representing less than 3% of all other insect species caught. This situation suggests that these flower strips were not attractive to thrips and may not serve as effective trap plants in this case. The effectiveness of flower strips in attracting natural enemies and providing significant biological control relies heavily on flower species selection [58]. Functional flower traits, such as corolla depth, petal color and flower phenology, are frequently used to favour the presence of specific insect groups [59,60]. To transfer these findings to other contexts and achieve a similar effect with floral resources, it is necessary to have a good understanding of the role of flower species selection and the response of the species of natural enemies present in the area. Furthermore, it should be noted that the size of the flower strips in our study was significant compared to that of the onion plot. The transfer of this technique to commercial fields will require large-scale retesting to validate the effectiveness of a smaller flower strip in protecting a larger onion area.

The beneficial effects of cover crops and mulching on agricultural production are widely recognized. However, the extent and nature of the effects of mulching vary significantly across different soils and plant groups [61,62,63,64]. In the context of vegetable production, mulching primarily serves to mitigate soil losses by limiting erosion and increasing organic matter inputs [65]. Mulches also act as a valuable ally against weeds by inhibiting their emergence both physically and chemically [66]. In this study, the use of straw mulch in 2021 demonstrated a beneficial effect by reducing onion thrips densities by 50% and increasing marketable yield by approximately 25%. These findings align with the research done by Larentzaki et al. [30] in New York State, which showed a significant reduction in *T. tabaci* adult and larval populations with the use of straw mulch without adverse effects on onion yield. Similar observations were made in a study conducted in Colorado, which reported that onion thrips abundance decreased by as much as 45% and also contributed to reduced Iris yellow spot virus incidence and severity [67]. However, in the present study in 2022, straw mulch had a less pronounced effect on onion thrips densities across both trial sites. The presence of mulch, particularly when combined with flowering strips, appeared to favour only yield. This suggests a cumulative effect of the two habitat manipulation techniques, which worked together to enhance biological control of onion thrips. However, as shown by Larentzaki [30], while ground predatory fauna, assessed through pitfall trapping, did not increase with straw mulch, neither did the population of the common predatory thrips *Aeolothrips fasciatus* (L.) (Thysanoptera: Aeolothripidae). Instead, the lower number of thrips on onions grown on straw mulches could be explained by the interference of straw mulch in the pupation and emergence of *T. tabaci* from the covered soil. Additionally, it is noteworthy that the mulch used (switchgrass) was very pale in color and may have had an effect similar to reflective plastic mulches, which have demonstrated positive results for onion thrips control, possibly due to visual deterrence [31,68,69].

### 4.2. Biological Control Agents, Used Alone or in Combination

The use of biological control agents in open-field horticultural crops presents several challenges which relate to environmental variability, crop diversity, pest mobility, pesticide use, landscape context, and economic factors [70,71]. These factors can affect the efficacy of biological control agents and hinder the adoption of biocontrol approaches by growers.

A limited number of studies have investigated the efficacy of ground predatory mites as a biological control agent against onion thrips in greenhouse or field production. Wu et al. [72] showed that the use of *S. scimitus* to control *T. tabaci* on greenhouse cucumbers was effective, achieving a 64% reduction in the pest population. Another study [73] demonstrated the effectiveness of *S. scimitus* in controlling western flower thrips (*Frankliniella* spp.), with a reduction of up to 75% in population density. In addition, in a greenhouse trial on the control of the western flower thrips (*F. occidentalis*) on eggplant, the use of the predatory mite *S. scimitus* led to decreases of about 73% in adult thrips and 66% in larvae [74]. In our 2021 trial, we did not observe differences in onion thrips control between the *S. scimitus* and the control including insecticide use at the AAFC site, not even when two to four applications of this mite species were made during the growing season. Other biocontrol treatments, including the combined use of *S. scimitus*, *N. cucumeris* and *B. bassiana*, resulted in similar thrips densities to the other control strategies at the AAFC site. Furthermore, on both commercial sites, *S. scimitus* was applied in combination with *N. cucumeris* or with the addition of *B. bassiana*, but none of these combination strategies significantly reduced onion thrips populations relative to the control. Very few studies involving the use of *S. scimitus* have been conducted in open-field crops, and it is possible that this species is poorly suited for use in onion production, where temperatures can be very high and optimal shelters (i.e., shade under the leaves) are not available for arthropod thermoregulation. The 2021 season was characterized by very hot and dry conditions, which may explain why this biological control agent failed to establish in the field. Furthermore, the mite densities applied in our field trial may have been an issue, since a lower density was applied (50 mites/m^2^) than the densities (between 250 and 1000 mites/m^2^) used under greenhouse conditions in other studies [17,73]. The aim of this low-density approach in the open-field production context was to allow the biological control agents to establish and multiply, so as to provide adequate control of thrips while minimizing the economic costs associated with the introduction of *S. scimitus*.

Biological control of onion thrips and western flower thrips has also been carried out using foliar predatory mites, such as *N. cucumeris* and *A. swirskii* [75,76,77]. *Neoseiulus cucumeris* is a biological control agent that is commonly used to efficiently control thrips species (mainly *F. occidentalis*) in greenhouse-based cut flower production [77]. In the present study, combined inoculations of *N. cucumeris* and *B. bassiana* or *S. scimitus* failed to control thrips populations in 2021. It has often been conjectured that the low efficiency of this predatory mite is due to its poor performance at temperatures higher than 25 °C and in dry conditions [75,78]. The predatory mite *N. cucumeris* is well adapted to pollen-producing crops such as sweet pepper, given that pollen can serve as an alternative food resource when prey is scarce [79]. This may explain its poor performance in onions. *A. swirskii* is better adapted to greenhouse conditions, including hot and dry climates, while *N. cucumeris* is not well suited to these conditions [76]. For this reason, *A. swirskii* was assessed in onion fields in 2022, but it did not provide additional control over onion thrips populations. In the literature, there are contradictory results regarding the nutritional value of *T. tabaci* for *A. swirskii*. One study [76] suggests that compared to other food sources like pollen, mixed diets, and white flies, the nutritional value of *T. tabaci* is low and does not support the survival and reproduction of *A. swirskii*. However, another study [80] proposes that onion thrips are an excellent food source for *A. swirskii*, which prefers to prey on onion thrips over western flower thrips. In the present study, although thrips densities were not significantly different from the untreated control, the use of *A. swirskii* alone or in combination with *B. bassiana* seemed to cause a slight decrease in CTD on some sites in 2022. Additional research with the aim of optimizing the application frequency and doses of these biological control agents would assist in validating their effectiveness.

The effectiveness of the entomopathogenic fungus *B. bassiana* in controlling onion thrips was inconsistent across the various trials. A significant reduction in thrips populations on onion leaves was not observed at all sites, and an unexpected decline in yield occurred at Site 1 in 2021. To maintain the continuous presence of *B. bassiana* spores on onion leaves, the fungus was applied multiple times (ranging from two to six applications) throughout the growing season. Nonetheless, the survival of *B. bassiana* can be adversely affected by abiotic factors such as high temperatures, dry conditions, and UV exposure [48]. Wu et al. [72] noted that the effect of *B. bassiana* treatments on *T. tabaci* mortality usually becomes apparent after one week. However, this dynamic may vary in field conditions, where climatic factors could compromise spore persistence on leaves, potentially reducing thrips control efficacy. Since thrips prepupae and pupae are stationary soil-dwelling stages and they are particularly vulnerable to soilborne pathogens, Ansari et al. [81] suggested that research be conducted to explore the potential of delivering entomopathogenic treatments directly to the soil rather than relying on foliar applications.

In situations where pest populations are high, relying on a single biocontrol agent may prove insufficient for achieving effective control. Additional measures are therefore often necessary, such as the use of chemical sprays (which may interfere with biocontrol efforts) or a combination of natural enemies to prevent detrimental pest population growth. In this trial against onion thrips, *B. bassiana* was selected primarily as an adjunct to be used with another biological control agent (*N. cucumeris* or *A. swirskii*). This innovative approach of combining a predatory mite with an entomopathogenic fungus has shown potential for enhancing pest population control [45]. A predatory mite that specializes in consuming young thrips larvae and can easily navigate among leaves, is an ideal agent for targeting developing thrips populations. Moreover, by acting as a vector for *B. bassiana* spores, such a mite can deliver the fungus to infested areas and control adult thrips. In our study, no advantages were observed when different biological control agents were applied simultaneously, such as the use of a foliar predatory mite inoculated with *B. bassiana* or a combination of two predatory mites (foliar and soil predatory mites) with *B. bassiana*. Jacobson et al. [82] reported improved thrips control in pepper from the combined use of the foliar-dwelling predatory mite *N. cucumeris* and *B. bassiana*, with no significant adverse effects of the pathogen on the predatory mites. However, other studies have produced conflicting findings. For example, Manners et al. [77] found that there was no thrips control advantage to simultaneously using different foliage and soil biological control agents. The use of multiple agents can sometimes lead to negative interactions, such as intraguild predation, where biological control agents consume one another instead of the target pest [83,84]. Careful selection of biological control agents is crucial in order to avoid detrimental effects such as these [85,86]. Further research should be undertaken to validate the parameters that would ensure that individual biological control agents are effective in managing onion thrips, before efforts are devoted to exploring the possibility of combining different agents.

### 4.3. Products Aimed at Reducing Plant Colonization by Thrips

Some studies have demonstrated that a kaolin-based particle film applied to onion leaves has potential as a pest management strategy because it reduces pest insects from colonizing crop plants [30]. For example, kaolin particle film had a significant inhibitory effect on thrips feeding behavior, as evidenced by a 16% decrease in thrips colonization of kaolin-treated onion leaves relative to untreated leaves [87]. In our study, the use of kaolin did not improve thrips control, in spite of the fact that more than eight applications were made during the growing season. In addition, there was no improvement in yield, although an increasing yield trend was observed at Site 1 in 2022. Similar results have been obtained with the use of mineral oil, a product applied to suffocate insects, prevent feeding, and limit thrips colonization of plants [39]. However, other studies that tested mineral oils found significant decreases in pest abundance; for example, a decrease of up to 78% in the number of bean thrips (*Megalurothrips distalis* [Karny]) (Thysanoptera: Thripidae) was found in summer mung bean in India [88]. The differences in climatic conditions from one region of the world to another may explain the differences in response. In the present study, frequent precipitation events during the growing season in 2022 may have had a significant impact on the efficacy of kaolin and mineral oil for protecting onion crops. Taking into account these findings, repeated and costly applications of such products may not be a viable strategy for onion growers. It may be more effective to use kaolin or mineral oil during specific periods of the production cycle, for instance, at the start of the season to mitigate early colonization of thrips, or towards the end of the season, when high temperatures may make it inadvisable to apply biological control agents in the field.

Exclusion nets, which serve as physical barriers, are gaining popularity in agriculture as an eco-friendly means of controlling insect pests which can reduce the reliance on chemical pesticides to protect crops from damage [40]. However, in our study, onions produced under these nets were smaller, and the overall yield was significantly reduced. The prolonged use of nets throughout the 3–4-month onion production cycle poses challenges, given the need to periodically remove the nets for weeding and disease management, which gives thrips the opportunity to colonize plants. Very fine mesh sizes (0.25 × 0.35 mm) were used to exclude *T. tabaci*. Paradoxically, however, these nets contributed to disease development by increasing humidity and temperature as well as friction injuries. Similar adverse effects on plant growth were observed with a 0.4 mm mesh net applied over cruciferous vegetables [89]. In another study, a mesh size of 0.6 mm was found to be insufficient to completely exclude thrips from cruciferous vegetables [90]. Alternative strategies have also been explored, such as using colored exclusion screens to reduce thrips’ ability to recognize host plants by exploiting their limited perception of certain wavelengths in red tones [91]. This control technique would need to be adapted to effectively manage thrips in vegetable crops without hindering plant growth.

## Data Availability

All relevant data are presented in the paper. Additional data can be supplied upon request.

## Supplementary Materials

The following supporting information can be downloaded at: https://www.mdpi.com/article/10.3390/insects15040232/s1, Figure S1: Seasonal variation in the mean number of Thrips tabaci per plant (±SE) in onion fields in 2021 at A) Site 1; B) Site 2 and C) AAFC site; Figure S2: Seasonal variation in the mean number of Thrips tabaci adults per plant (±SE) in onion fields in 2021 at A) Site 1 and B) Site 2 (data not available on Site AAFC); Figure S3: Seasonal variation in the mean number of Thrips tabaci juveniles per plant (±SE) in onion fields in 2021 at A) Site 1 and B) Site 2 (data not available on Site AAFC); Figure S4: Seasonal variation of the mean number of Thrips tabaci per leaf (±SE) in onion fields in 2022 at A) Site 1; B) AAFC site; Figure S5: Seasonal variation in the mean number of Thrips tabaci adults per plant (±SE) in onion fields in 2022 at A) Site 1 and B) AAFC Site; Figure S6: Seasonal variation in the mean number of Thrips tabaci juveniles per plant (±SE) in onion fields in 2022 at A) Site 1 and B) AAFC Site; Figure S7: Correlation between cumulative thrips-days and onion yield 2021 for all sites; Figure S8: Correlation between cumulative thrips-days and onion yield 2022 from all sites; Figure S9: Relative abundance of insect biodiversity sampled by sweep net in flower strips during the 2021 season; Table S1: Date of sowing or transplanting of the different dry onion cultivars on the three experimental sites; Table S2: Description of herbicide and fungicide treatments and date of application on the three experimental sites, in 2021 and 2022; Table S3: Date of application and type of biological control strategy (or insecticides) applied to control Thrips tabaci in dry onion in 2021; Table S4: Date of application and type of biological control strategy (or insecticides) applied to control Thrips tabaci in dry onion in 2022; Table S5: Grades of onions in 2021 on the different experimental sites; Table S6: Grades of onions in 2022 on the different experimental sites; Table S7: Total abundance of predators observed on onions during the monitoring period, for the entire season.
