# Supplementary material for: Biological Control and Habitat Management for the Control of Onion Thrips, Thrips tabaci Lindeman (Thysanoptera: Thripidae), in Onion Production in Quebec, Canada
Source: Insects. 2024 Mar 27;15(4):232. doi: 10.3390/insects15040232 (PMC11050518; doi:10.3390/insects15040232)
Supplement: Supplementary file 1 [file insects-15-00232-s001.zip › insects-2857622-supplementary.pdf]

Biological Control and Habitat Management for the Control of Onion Thrips, *Thrips tabaci* Lindeman (Thysanoptera: Thripidae), in Onion Production in Quebec, Canada

Supplemental information

Supplemental Table S1. Date of sowing or transplanting of the different dry onion cultivars on the three experimental sites.

	AAFC site	Site 1	Site 2
2021	Trailblazer[1], Sown on May 4	Patterson[2], Planted on May 26	Cartier[3], Sown on May 12
2022	Trailblazer, Sown on May 3	Patterson, Planted on May 31	-

[1] Yellow onion, maturity = 94 days, high resistance to *Botrytis* spp., intermediate resistance to pink root.
[2] Yellow onion, maturity = 105 days, intermediate resistance to pink root.
[3] Yellow onion, maturity = 94 days, intermediate resistance to pink root.

Supplemental Table S2. Description of herbicide and fungicide treatments and date of application on the three experimental sites, in 2021 and 2022.

Site	2021		2022	
	Treatments	Dates	Treatments	Dates
AAFC site	Frontier (herbicide)	7 May	Frontier (herbicide)	5 May
	Prowl (herbicide)	20 May	Prowl (herbicide)	21 May
	Prowl (herbicide)	12 June	Venture (herbicide)	4 June
	Manzate (fungicide)	3 July	Prowl (herbicide)	11 June
	Sercadis (fungicide)	9 July	Quadris (fungicide)	15 July
	Manzate (fungicide)	16 July	Revus (fungicide)	16 July
	Fontelis (fungicide)	24 July	Quadris (fungicide)	22 July
			Ridomil gold (fungicide)	23 July
			Manzate (fungicide)	29 July
			Manzate (fungicide)	5 August
Site 1	Serenade Opti (fungicide)	9 August	Serenade Opti (fungicide)	22 July
			Copper Spray (fungicide)	6 August
			Copper Spray (fungicide)	15 August
Site 2	Venture (herbicide)	1 June		
	Prowl (herbicide)	4 June		
	Pardner (herbicide)	12 June		
	Manzate (fungicide)	17 June		
	Manzate (fungicide)	24 June		
	Select (herbicide)	28 June		
	Prowl (herbicide)	8 July		
	Orondis Ultra (fungicide)	12 July		
	Luna Tranquility (fungicide)	21 July		
	Reason 500 SC (fungicide)	28 July		

Supplemental Table S3. Date of application and type of biological control strategy (or insecticides) applied to control *Thrips tabaci* in dry onion in 2021.

Site	Biological control agent	No. of applications	Dates of application
**AAFC site**	*Stratiolaelaps scimitus*	3	14 June; 5, 26 July
	Neoseiulus cucumeris + Beauveria bassiana	3	11 June 11; 5,26 July
	Stratiolaelaps scimitus + Neoseiulus cucumeris + Beauveria bassiana	3	11 June 11; 5,26 July
	Movento (spirotetramat)	2	12, 18 June
	Agrimek (abamectin)	2	25 June; 2 July
	Exirel (cyantraniliprole)	2	9, 16 July
**Site 1**	*Stratiolaelaps scimitus*	4	11, 30 June; 22 July; 12 Aug.
	Neoseiulus cucumeris + Beauveria bassiana	4	11, 30 June; 22 July; 12 Aug.
	Stratiolaelaps scimitus + Neoseiulus cucumeris + Beauveria bassiana	4	11, 30 June; 22 July; 12 Aug.
	BioCérès® (*Beauveria bassiana*)	6	1, 13, 22, 30 July; 5, 16 Aug.
	Entrust (spinosad)	3	1, 13, 30 July
**Site 2**	*Stratiolaelaps scimitus*	2	11, 30 June
	Neoseiulus cucumeris + Beauveria bassiana	2	18 June, 9 July
	Stratiolaelaps scimitus + Neoseiulus cucumeris + Beauveria bassiana	2	11, 30 June (*S. scimitus*)<br>18 June, 9 July (*N. cucumeris + B. bassiana*)
	BioCérès® (*Beauveria bassiana*)	3	7, 28 July; 5 Aug.
	Movento (spirotetramat)	1	7 July
	Agrimek (abamectin)	1	28 July
	Exirel (cyantraniliprole)	1	5 August

Supplemental Table S4. Date of application and type of biological control strategy (or insecticides) applied to control *Thrips tabaci* in dry onion in 2022.

Site	Biological control agent	No. of applications	Dates of application
AAFC site	*Amblyseius swirskii*	2	24, 30 June
	BioTitan® (*Beauveria bassiana*)	2	25 June, 13 July
	Amblyseius swirskii + *Beauveria bassiana*	2	24, 30 June
	Surround®	7	14, 25 June; 1, 6, 13, 22, 29 July
	SuffOil-X®	7	14, 25 June; 1, 6, 13, 22, 29 July
Site 1	*Amblyseius swirskii*	8	24, 30 June; 14, 22, 28 July; 4, 11, 18 Aug.
	BioTitan® (*Beauveria bassiana*)	6	28 June; 6, 13, 19, 29 July; 5 Aug.
	Amblyseius swirskii + *Beauveria bassiana*	8	24, 30 June; 14, 22, 28 July; 4, 11, 18 Aug.
	Surround®	9	28 June; 6, 13, 20, 27 July; 3, 11, 17, 25 Aug.
	SuffOil-X®	9	28 June; 6, 13, 20, 27 July; 3, 11, 17, 25 Aug.

Supplemental Table S5. Grades of onions in 2021 on the different experimental sites.

Site	Habitat manipulation	Biological control agent	> 7.62 cm	6.99–7.62 cm	6.36–6.98 cm	5.72–6.35 cm	5.08–5.71 cm	Downgraded (<5.08 cm)
AAFC site	Bare ground	*N. cucumeris + B. bassiana*	0.31a*	7.36a	22.75ab	32.52a	16.48a	20.59b
		S. scimitus	0.00a	1.09a	19.15a	41.46a	18.28a	14.56ab
		S. sc +N. cu + B. ba	0.67a	7.27a	21.35a	28.24a	21.75a	20.73b
		insecticides	0.58a	11.37a	34.66b	33.82a	12.69a	6.89a
	Straw mulch	*N. cucumeris + B. bassiana*	0.58a	9.49a	32.86a	30.73a	15.05a	11.29a
		S. scimitus	1.14a	11.62a	34.51a	32.19a	12.47a	8.07a
		S. sc +N. cu + B. ba	0.28a	7.59a	32.86a	35.21a	16.41a	12.33a
		insecticides	1.30a	13.34a	40.16a	30.55a	9.93a	4.73a
	Flower strip	*N. cucumeris + B. bassiana*	0.00a	6.55a	29.98a	23.25a	27.25a	12.97a
		S. scimitus	0.54a	10.01a	34.11a	25.68a	19.06a	10.59a
		S. sc +N. cu + B. ba	0.28a	5.95a	34.31a	37.32a	14.31a	7.84a
		Insecticides	0.79a	7.07a	39.57a	31.90a	11.94a	8.71a
Site 1	Bare ground	Untreated control	0.00a	2.09a	17.09a	33.99a	26.32a	19.82a
		N. cucumeris + B. bassiana	0.00a	2.22a	12.99a	38.66a	30.41a	14.66a
		N. cucumeris + S. scimitus	0.40a	2.30a	12.14a	31.55a	31.50a	20.90a
		N. cucumeris + B. bassi + S. sci	0.00a	3.02a	24.15a	29.51a	24.54a	17.62a
		B. bassiana	0.37a	4.33a	9.90a	27.94a	22.92a	34.16a
		Insecticides	0.00a	5.69a	20.41a	32.57a	26.05a	13.45a
Site 2	Bare ground	Untreated control	0.17a	1.18a	10.13a	33.96a	29.23a	24.62a
		N. cucumeris + B. bassiana	0.00a	0.57a	12.66a	31.40a	32.25a	22.73a
		N. cucumeris + S. scimitus	0.18a	1.82a	13.48a	32.52a	25.86a	24.88a
		N. cucumeris + B. bassi + S. sci	0.00a	1.51a	9.87a	36.73a	27.06a	23.37a
		B. bassiana	0.00a	0.78a	9.53a	34.79a	32.13a	21.08a
		Insecticides	0.00a	0.73a	12.23a	33.65a	31.30a	21.05a

*Statistical comparisons were conducted between each of the treatments for the same onion grade. Different letters indicate values that are significantly different according to ANOVA followed by a Tukey test (p<0.05).

Supplemental Table S6. Grades of onions in 2022 on the different experimental sites.

Site	Treatments	> 7.62 cm	6.99–7.62 cm	6.36–6.98 cm	5.72–6.35 cm	5.08–5.71 cm	Downgraded (<5.08 cm)
AAFC Site	Mulch	1.51b	12.59bc	33.56a	24.95a	12.68bc	14.71bcd
	Net	0.38b	0.82c	11.36c	25.71a	35.26a	26.48a
	B. bassiana	3.42b	6.13bc	16.79bc	24.56a	24.41ab	24.68ab
	A. swirskii	21.87a	28.66a	19.22bc	12.27a	6.47c	11.49d
	A. swirskii + B. bassi	6.01b	20.30ab	27.30ab	22.81a	12.12bc	11.46d
	Kaolin	3.62b	13.00abc	23.75abc	25.81a	12.84bc	20.98abcd
	Mineral oil	6.32b	18.72ab	22.32abc	22.49a	17.45bc	12.71cd
	Untreated control	2.35b	8.20bc	23.82abc	21.06a	20.83b	23.74abc
Site 1	Mulch	0.19b	1.13b	13.03abc	27.79a	22.76a	33.88bc
	Net	0.00b	0.00b	0.19c	4.39b	16.69a	78.35a
	B. bassiana	0.65ab	3.83b	14.81abc	27.21a	21.49a	30.47bc
	A. swirskii	3.17a	11.71a	26.39a	29.81a	12.82a	13.52c
	A. swirskii + B. bassi	1.91ab	3.70b	21.89ab	29.27a	21.00a	18.69bc
	Kaolin	0.19b	1.60b	8.53bc	21.81a	22.69a	38.83bc
	Mineral oil	0.65ab	4.59b	18.21ab	33.05a	24.64a	16.18bc
	Untreated control	0.00b	1.10b	7.46bc	20.56ab	25.05a	44.45b

*Statistical comparisons were conducted between each of the treatments for the same onion grade. Different letters indicate values that are significantly different according to ANOVA followed by a Tukey test (p<0.05).

Supplemental Table S7. Total abundance of predators observed on onions during the monitoring period, for the entire season.

Year	Site	Treatment	Total number of predators*	Total number of predator per site
2021	Site 1	Insecticide	4	82
		No insecticide	17	
		N. cucumeris + *B. bassiana*	14	
		N. cucumeris + *S. scimitus*	20	
		B. bassiana	14	
		S. scimitus + *N. cucumeris* + *B. bassiana*	13	
	Site 2	Insecticide	8	53
		No insecticide	10	
		N. cucumeris + *B. bassiana*	11	
		N. cucumeris + *S. scimitus*	15	
		B. bassiana	4	
		S. scimitus + *N. cucumeris* + *B. bassiana*	5	
	AAFC	Bare ground	5	17
		Mulch	9	
		Flowers	3	
2022	Site 1	No insecticide	77	427
		Mulch	79	
		Kaolin	23	
		Mineral oil	56	
		A. swirskii	61	
		B. bassiana	64	
		A. swirskii + *B. bassiana*	67	
	AAFC	No insecticide	8	44
		Mulch	3	
		Mulch+flowers	8	
		Flowers	4	
		Kaolin	7	
		Mineral oil	1	
		A. swirskii	2	
		B. bassiana	8	
		A. swirskii + *B. bassiana*	3	

*Predators included Coccinellidae, Syrphidae, Aeolothripidae and Chrysopidae.

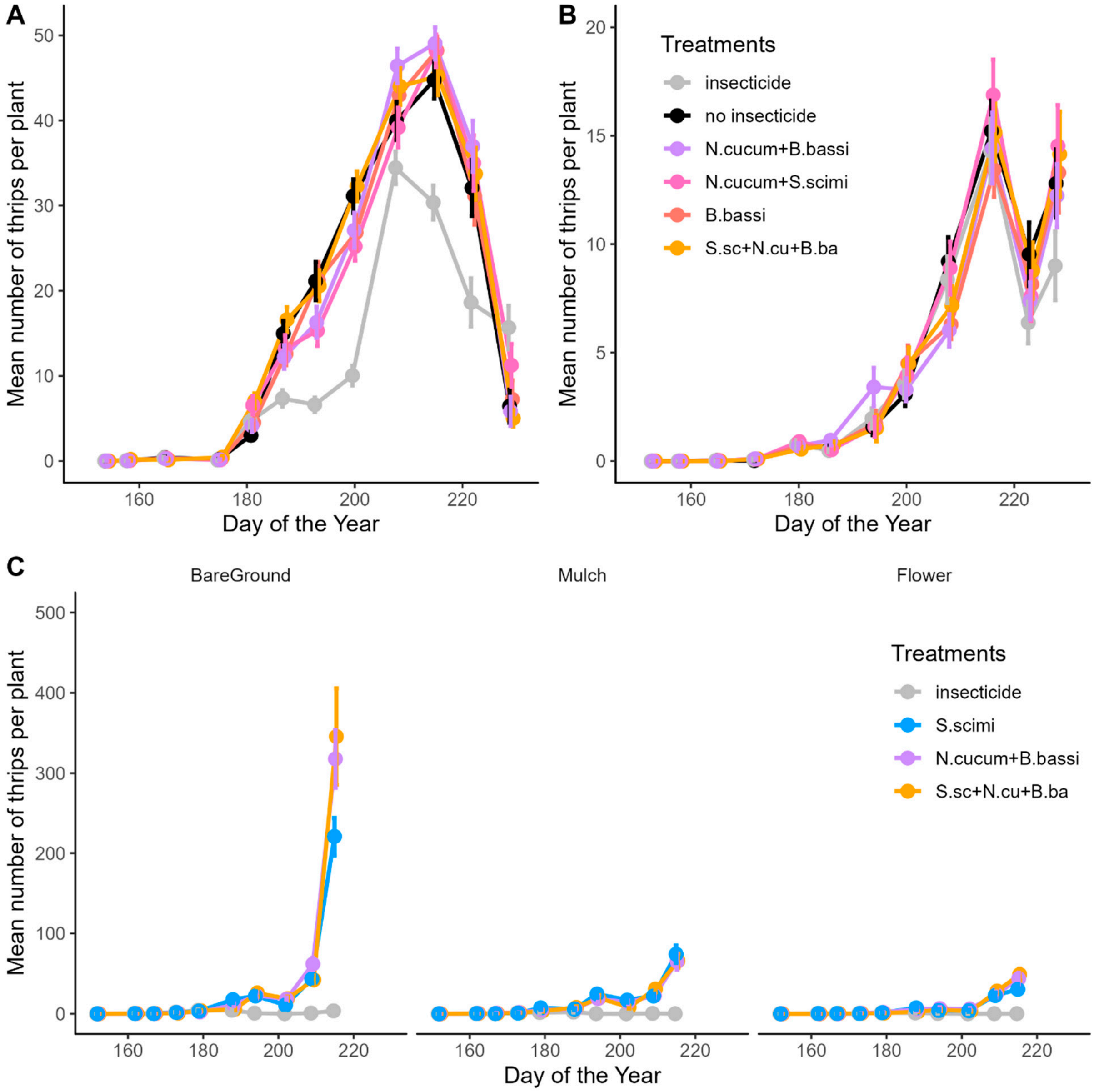

Supplemental Figure S1. Seasonal variation in the mean number of *Thrips tabaci* per plant (±SE) in onion fields in 2021 at A) Site 1; B) Site 2 and C) AAFC site.

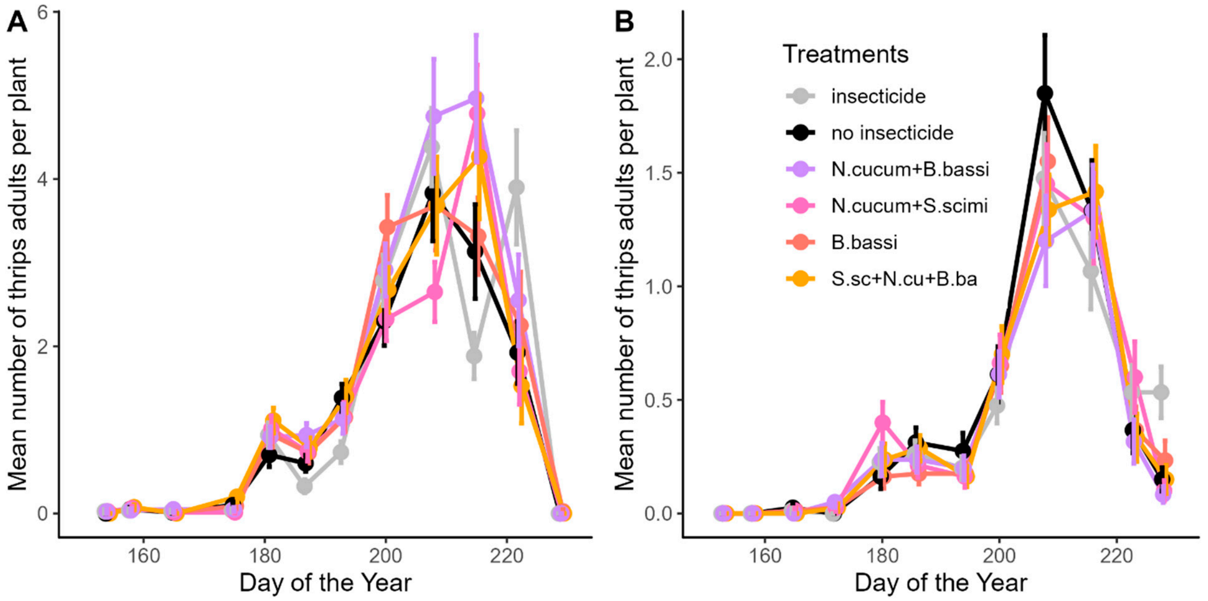

Supplemental Figure S2. Seasonal variation in the mean number of *Thrips tabaci* adults per plant (±SE) in onion fields in 2021 at A) Site 1 and B) Site 2 (data not available on Site AAFC).

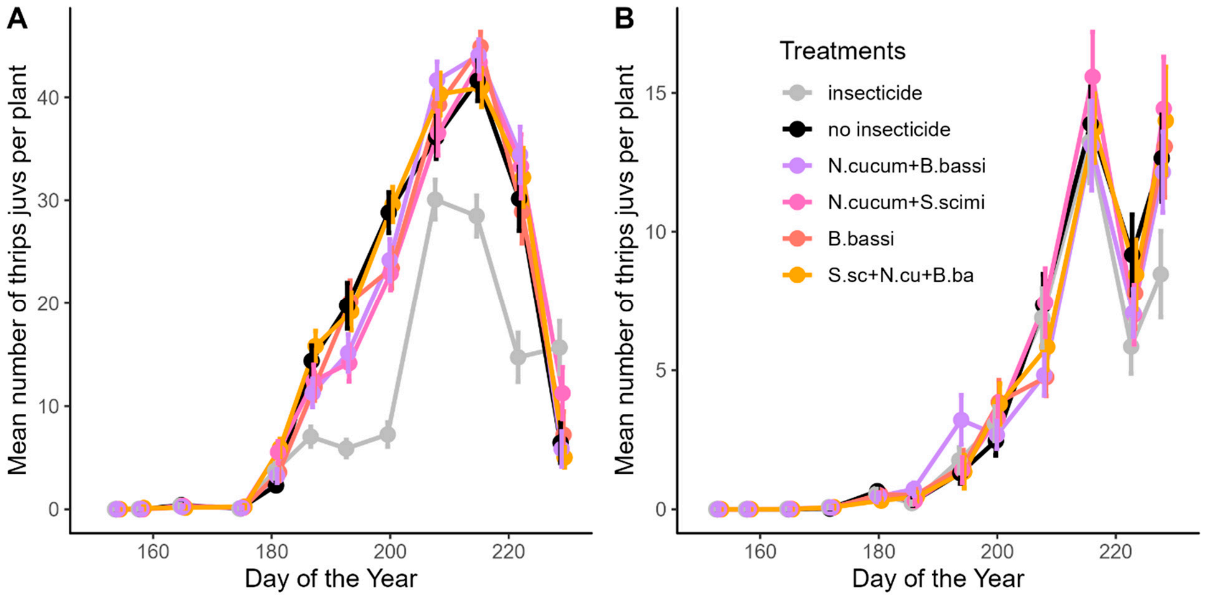

Supplemental Figure S3. Seasonal variation in the mean number of *Thrips tabaci* juveniles per plant (±SE) in onion fields in 2021 at A) Site 1 and B) Site 2 (data not available on Site AAFC).

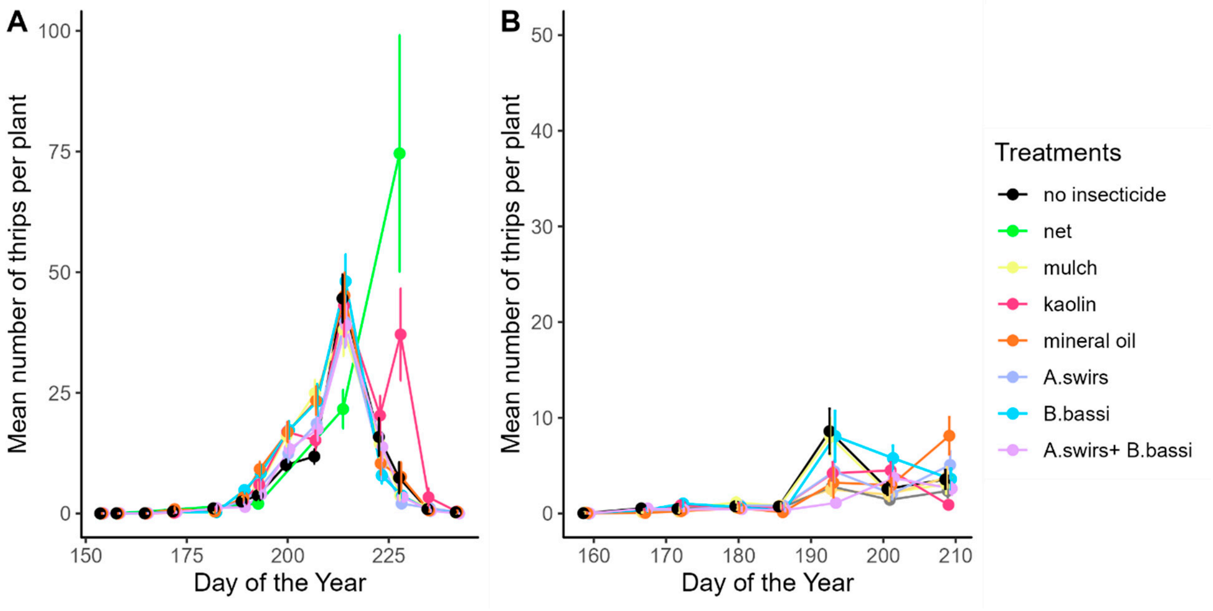

Supplemental Figure S4. Seasonal variation of the mean number of *Thrips tabaci* per leaf (±SE) in onion fields in 2022 at A) Site 1; B) AAFC site.

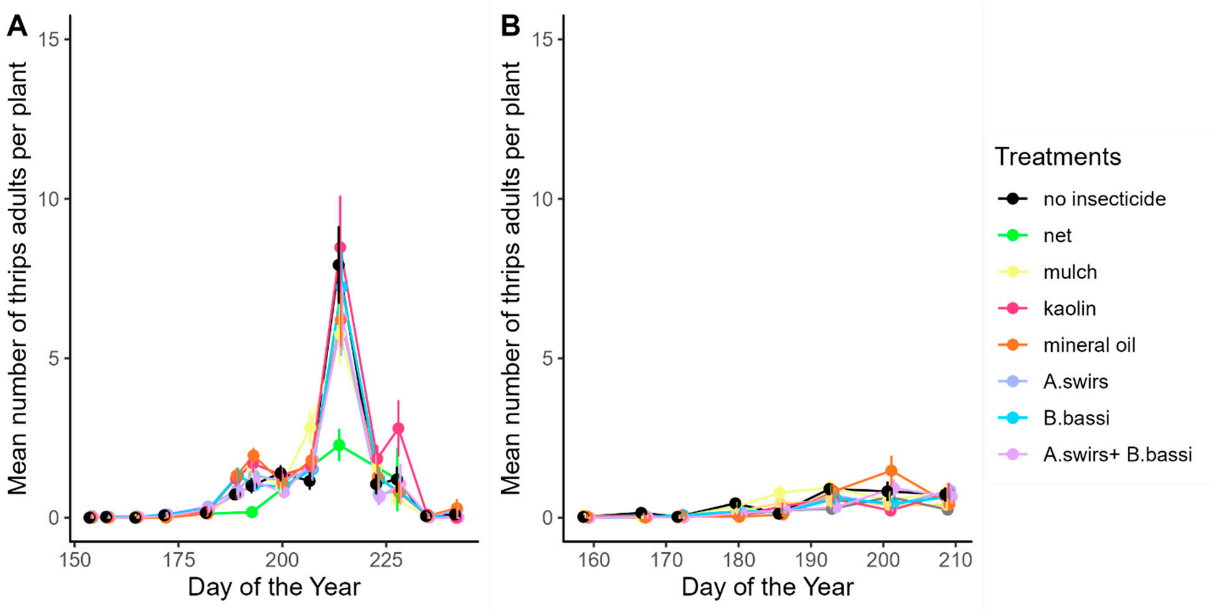

Supplemental Figure S5. Seasonal variation in the mean number of *Thrips tabaci* adults per plant (±SE) in onion fields in 2022 at A) Site 1 and B) AAFC Site.

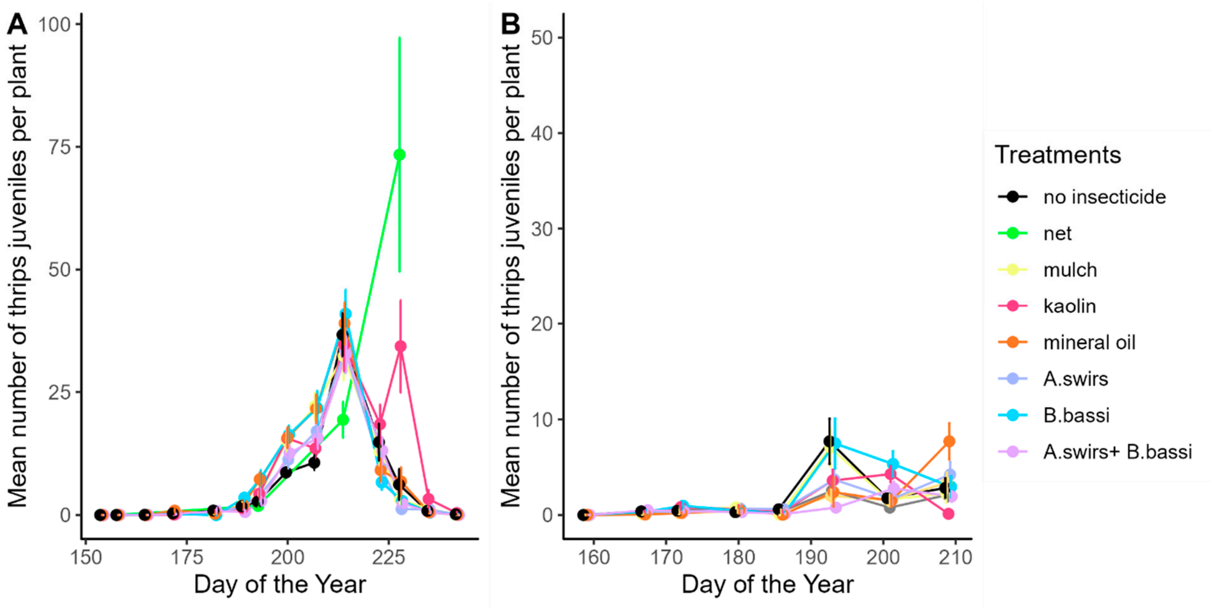

Supplemental Figure S6. Seasonal variation in the mean number of *Thrips tabaci* juveniles per plant (±SE) in onion fields in 2022 at A) Site 1 and B) AAFC Site.

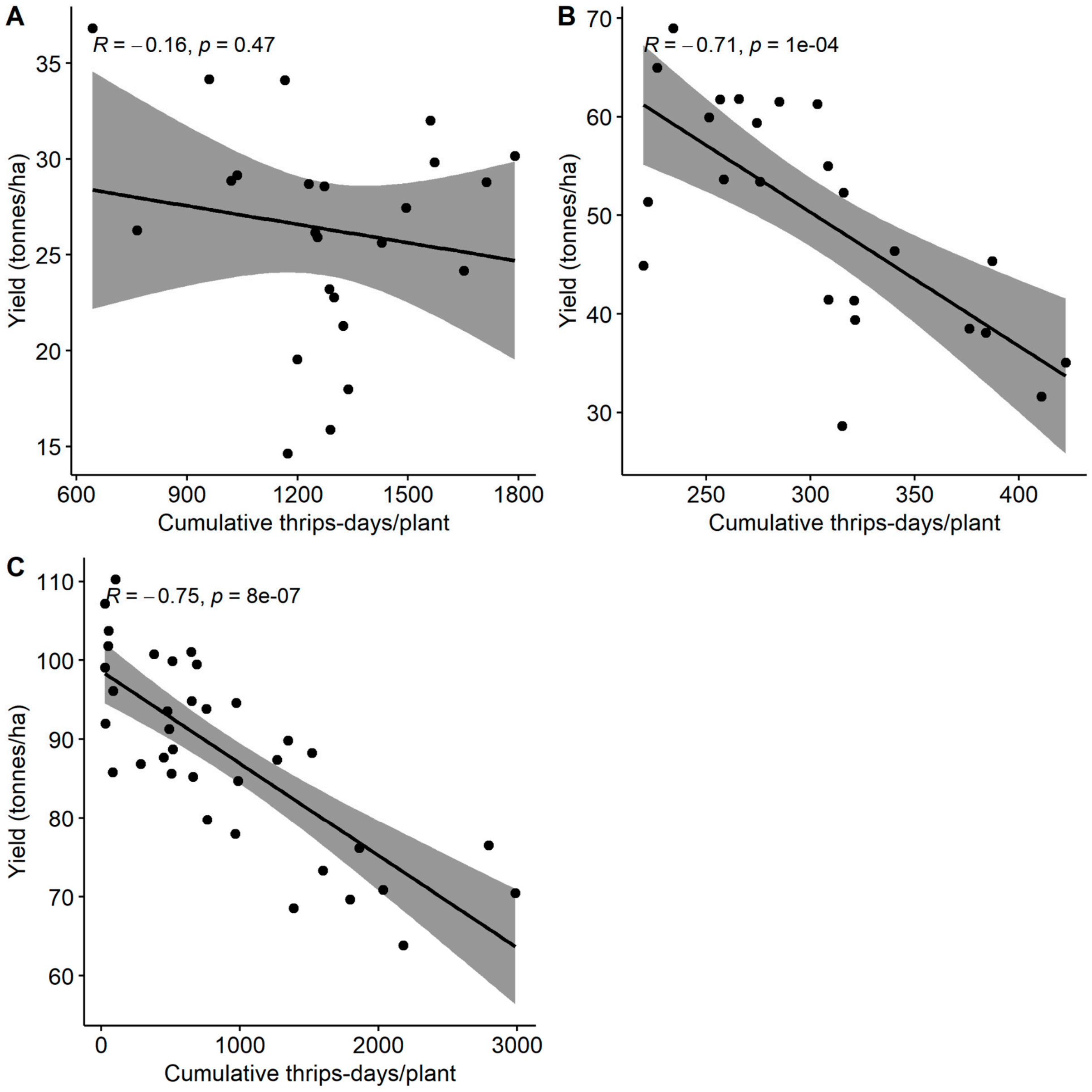

Supplemental Figure S7. Correlation between cumulative thrips-days and onion yield 2021 for all sites.

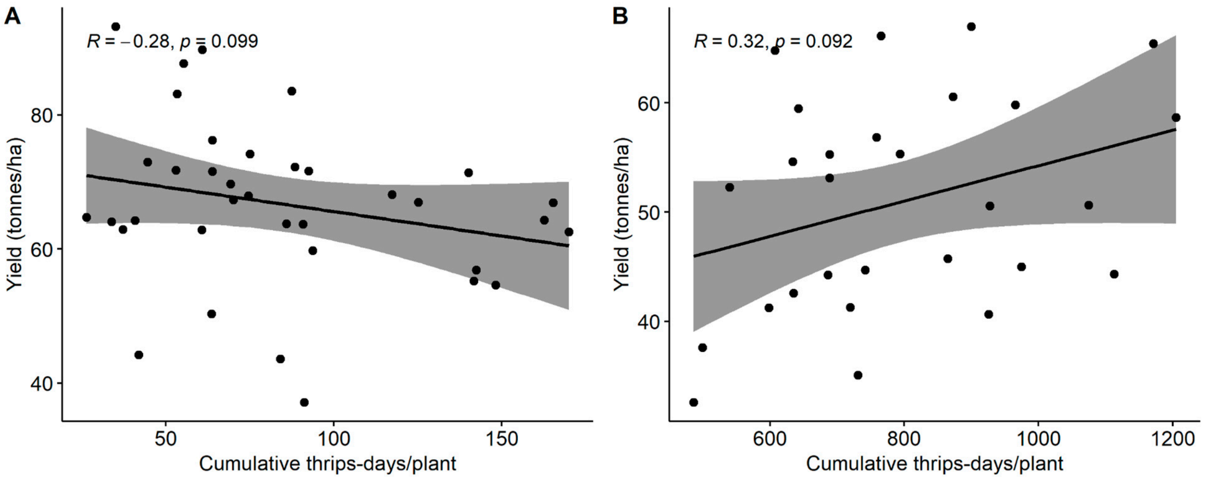

Supplemental Figure S8. Correlation between cumulative thrips-days and onion yield 2022 from all sites.

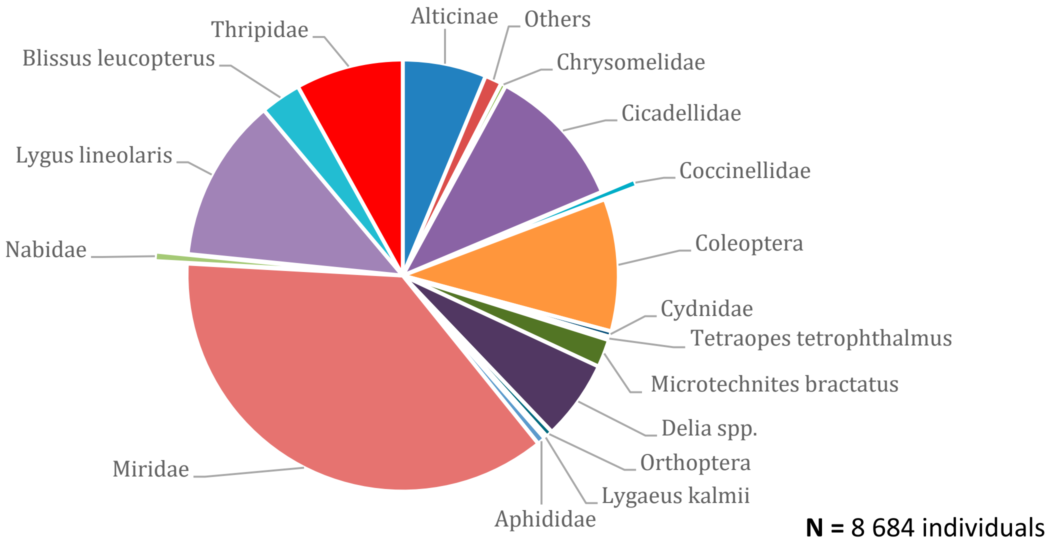

Supplemental Figure S9. Relative abundance of insect biodiversity sampled by sweep net in flower strips during the 2021 season.
